# THE ASSOCIATIONS OF OPIOID USE IN GUN-RELATED FATALITIES AMONG OLDER DECEDENTS IN FLORIDA

**DOI:** 10.1093/geroni/igad104.2690

**Published:** 2023-12-21

**Authors:** Armiel Suriaga, Ruth Tappen, David Newman

**Affiliations:** Florida Atlantic University, Boca Raton, Florida, United States; Florida Atlantic University, Boca Raton, Florida, United States; Florida Atlantic University, Boca Raton, Florida, United States

## Abstract

In 2020, the Center for Disease Control and Prevention reported the highest firearm deaths in the U.S. at 45, 222. Homicides and suicides accounted for most fatalities. However, the role of opioids in firearm-related deaths (FRD) among older adults was less explored. Our study aimed to investigate the associations between opioid use and FRD among people >65 years with opioids detected in their system at time of death. We used descriptive statistics to describe decedents’ characteristics using the 2021 Florida Department of Law Enforcement de-identified data. We used Pearson’s chi-square and logistic regression to examine the associations of opioids to FRD. All data were analyzed using Stata 17. A total of 1982 older decedents were included in the analysis. Age ranged from 65-100 years, mean 72.97 (SD=7.331). Three hundred thirty of 1982 deaths (16.65%) were gun-related; 121 of 330 (14.79%) had opioid involvement. More males (n=274) (83.03%) died than females; 316 (95.76%) were non-Hispanic whites, followed by black (n=10, 3.03%). Suicides accounted for most FRDs (n=330, or 90.91%), and homicide second (n=28 or 7.88%). The association between opioid use to FRD was statistically significant (p<.001). The odds of dying from FRD among older adults with any opioid use increased by .57 compared to those who never used any opioid, controlling for opioids combined with other substances, age, gender, and population density, OR=.57 (95% CI .41-.79). Our results have safety clinical implications as more older adults use opioids, e.g. for chronic pain, which calls for tailored interventions to protect older population.

